# Supplementary material for: An introduction to statistical models used to characterize species-habitat associations with animal movement data
Source: Mov Ecol. 2025 Apr 17;13:27. doi: 10.1186/s40462-025-00549-2 (PMC12004767; doi:10.1186/s40462-025-00549-2)
Supplement: Supplementary file 1 [file 40462_2025_549_MOESM1_ESM.docx]

# Appendix 1: Supplemental Figures and Tables


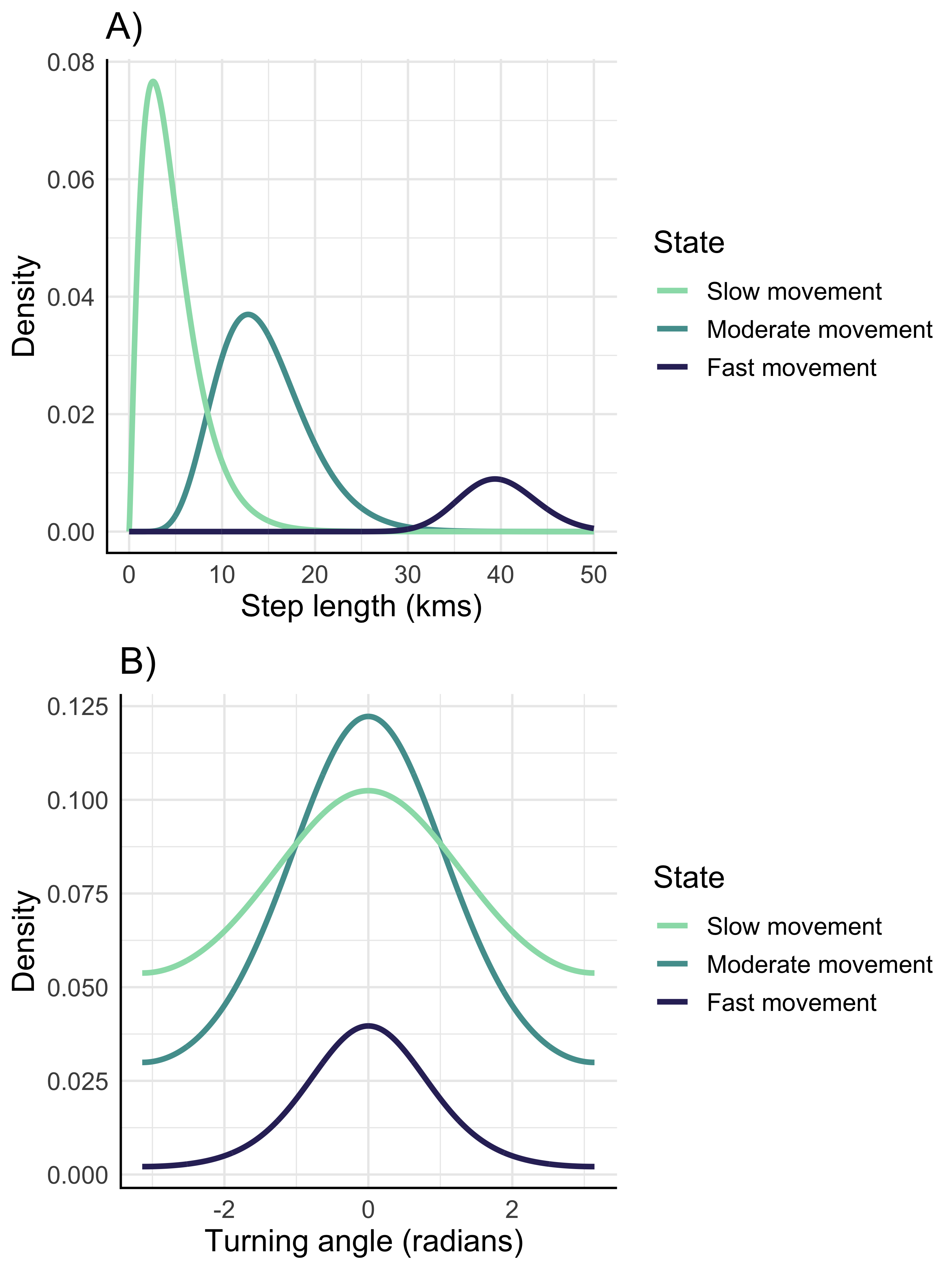


**Figure S1**. A) Step length and B) turning angle distributions of predicted states from the case study’s main HMM (i.e., the HMM featured in the main paper, where the covariate, prey diversity, only affects the transition probability matrix and not the observation probabilities, called hmm_trans_3 in the tutorial).


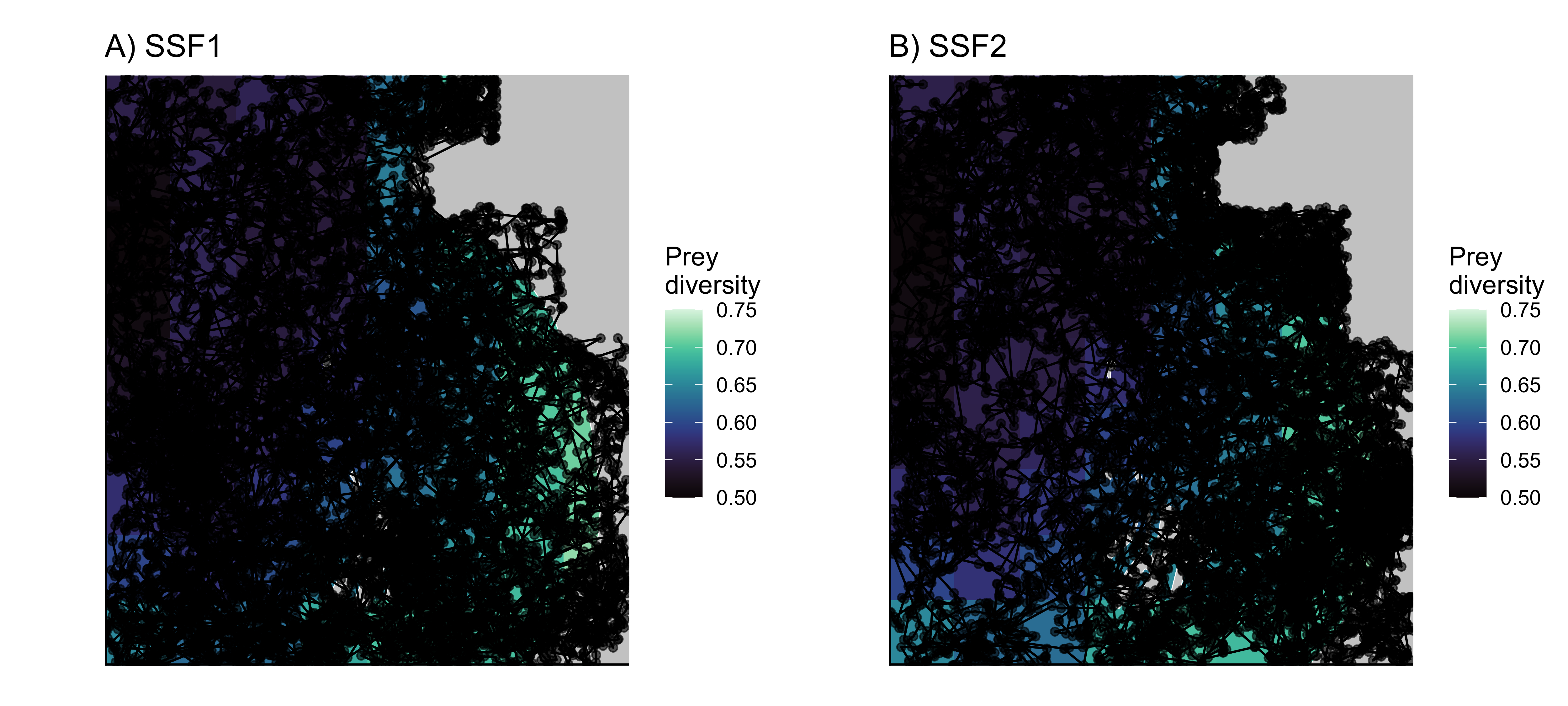


**Figure S2**. Simulated tracks (T = 10,000 locations) from the redistribution kernel that was generated from the step-selection functions where prey diversity A) without and B) with movement-related covariates. These simulated paths were used to generate a steady-state utilization distribution (SSUD) for each model. Note that we present here T = 10,000 locations (the sample size used in the tutorial) for visualization purposes. The SSUDs generated in Fig. 6 used T = 200,000 locations, which contained so many locations that they became indistinguishable from one another.

**Table S1**. Regression coefficients from the case study RSFs and SSFs.

| **Model** | **Parameter** | **ꞵ** | **SE** | **z-value** | **p-value** |
| --- | --- | --- | --- | --- | --- |
| **RSF on the full dataset** | | | | | |
|  | Intercept | -6.118 | 1.400 | -4.369 | <0.001 |
|  | Prey diversity | 6.353 | 2.748 | 2.748 | 0.006 |
| **RSF on the thinned dataset** | | | | | |
|  | Intercept | -5.763 | 4.397 | -1.310 | 0.190 |
|  | Prey diversity | 5.780 | 7.288 | 0.793 | 0.428 |
| **SSF without covariate affecting movement kernel (SSF1)** | | | | | |
|  | Prey diversity | 0.948 | 6.837 | 0.139 | 0.890 |
|  | Step length | <0.001 | <0.001 | -0.383 | 0.701 |
|  | Log (step length) | -0.042 | 0.157 | 0.269 | 0.788 |
|  | Cos (turning angle) | 0.031 | 0.131 | 0.235 | 0.814 |
| **SSF with covariate affecting movement kernel (SSF2)** | | | | | |
|  | Prey diversity | 31.500 | 23.090 | 1.364 | 0.173 |
|  | Step length | <0.001 | <0.001 | -0.645 | 0.519 |
|  | Log (step length) | 1.981 | 7.250 | 1.395 | 0.163 |
|  | Cos (turning angle) | 0.021 | 1.022 | 0.163 | 0.871 |
|  | Prey diversity: log (step length) | -3.123 | 2.253 | -1.386 | 0.166 |

ꞵ = parameter estimate, SE = standard error.

**Table S2**. Parameter estimates associated with the transition probability matrix of the case study’s main HMM (i.e., the HMM featured in the main paper, where the covariate, prey diversity, only affects the transition probability matrix, called hmm_trans_3 in the tutorial) regression coefficients for the transition probabilities, presented as “estimate (standard error)”, on the beta (i.e., working) scale of the parameters. Note that the standard error for the transition from state 1 to 3 is NA since this transition does not occur in our short example track; using a longer track would likely allow the model to estimate a standard error.

|  | **Estimate for transitioning from state*_t_* -> state*_t+1_*** | | | | | |
| --- | --- | --- | --- | --- | --- | --- |
| **Parameter** | **1 -> 2** | **1 -> 3** | **2 -> 1** | **2 -> 3** | **3 -> 1** | **3 -> 2** |
| Intercept | 11.958  (6.491) | -28.986  (<0.001) | 0.889  (6.070) | -6.008  (7.694) | 52.188  (0.892) | -12.384  (9.787) |
| Prey diversity | -22.386  (10.919) | 0.000  NA | -3.842  (9.974) | 6.299  (12.461) | -97.113  (0.562) | 19.088  (15.704) |

State 1 = slow movement, state 2 = moderate movement, state 3 = fast movement.

**Table S3**. Parameter estimates associated with the observation probability matrix of the case study’s main HMM (i.e., the HMM featured in the main paper, where the covariate, prey diversity, only affects the transition probability matrix, called hmm_trans_3 in the tutorial) including the regression coefficients for step length (mean and standard deviation [SD]) and turning angle (concentration), presented as “estimate (standard error)”, on the beta (i.e., working) scale of the parameters.

|  | **Step length** | | **Turning angle** |
| --- | --- | --- | --- |
| **State:Parameter** | **Mean** | **SD** | **Concentration** |
| Slow movement: Intercept | 1.544 (0.141) | 1.144 (0.203) | -1.133 (0.579) |
| Moderate movement: Intercept | 2.669 (0.056) | 1.581 (0.132) | -0.351 (0.321) |
| Fast movement: Intercept | 3.684 (0.030) | 1.424 (0.203) | 0.382 (0.379) |

**Table S4**. Parameter estimates associated with the transition probability matrix from the tutorial’s example of a HMM where the covariate, prey diversity, only affects the observation probability (i.e., not the transition probability matrix, called hmm_obs_3 in the tutorial), including regression coefficients for the transition probabilities, presented as “estimate (standard error)”, on the beta (i.e., working) scale of the parameters.

|  | **Estimate for transitioning from state*_t_* -> state*_t+1_*** | | | | | |
| --- | --- | --- | --- | --- | --- | --- |
| **Parameter** | **1 -> 2** | **1 -> 3** | **2 -> 1** | **2 -> 3** | **3 -> 1** | **3 -> 2** |
| Intercept | -1.709  (0.474) | -18.576 (0.119) | -1.692 (0.449) | -2.287 (0.506) | -16.157 (0.059) | -0.441 (0.586) |

State 1 = slow movement, state 2 = moderate movement, state 3 = fast movement.

**Table S5**. Parameter estimates associated with the observation probability matrix from the tutorial’s example of a HMM where the covariate, prey diversity, only affects the observation probability (i.e., not the transition probability matrix, called hmm_obs_3 in the tutorial), including the regression coefficients for step length (mean and standard deviation [SD]) and turning angle (concentration), presented as “estimate (standard error)”, on the beta (i.e., working) scale of the parameters.

|  | **Step length** | | **Turning angle** |
| --- | --- | --- | --- |
| **State:Parameter** | **Mean** | **SD** | **Concentration** |
| Slow movement: Intercept | 2.690 (1.318) | 1.082 (0.202) | 8.038 (7.387) |
| Slow movement: Prey diversity | -1.957 (2.125) | Not modeled | -15.016 (12.647) |
| Moderate movement: Intercept | 1.116 (0.674) | 1.611 (0.119) | -3.286 (4.846) |
| Moderate movement: Prey diversity | 2.495 (1.092) | Not modeled | 4.794 (7.899) |
| Fast movement: Intercept | 3.816 (0.411) | 1.455 (0.241) | 3.344 (4.104) |
| Fast movement: Prey diversity | -0.229 (0.678) | Not modeled | -4.922 (6.975) |

# Appendix 2: Full methods for case study on ringed seals in Hudson Bay

The data and code tutorial of analyses used for this case study are available as Appendix 2 and 3, respectively.

## Data collection and preparation

The seal, a two-year-old male (tag ID: 116484), was captured on 29 Oct 2012 using a monofilament mesh net set perpendicular to the shore along the coast of the Belcher Islands, Nunavut. We equipped the seal with a 9000x data logger from the Sea Mammal Research Unit (SMRU, University of St. Andrews, UK). See Florko et al., 2023 for details. We used one segment of the tracking data for the purpose of this case study, which had consistent transmissions over the course of 4.5 months.

We used estimated prey diversity using the estimated abundance of eight important ringed seal prey species: Arctic cod (*Boreogadus saida)*, capelin (*Mallotus villosus)*, northern sand lance (*Ammodytes dubius*), Pacific sand lance (*Ammodytes hexaoterus*), Arctic staghorn sculpin (*Gymnocanthus tricuspis*), shorthorn sculpin (*Myoxocephalus Scorpius*), mustache sculpin (*Triglops murrayi*), and rainbow smelt (*Osmerus mordax*) using Simpson’s Diversity Index (Florko et al., 2021a; 2021b, Fig 4). The fish data were modeled at a yearly time step on a 0.5^o^ longitude by 0.5^o^ degree latitude grid, under both a low emission (representative concentration pathway, RCP 2.6) and a high emission (RCP 8.5) emission scenario. We extracted the fish abundance data for each location in the corresponding grid cell. We used the high emission scenario as it aligned more closely to the emissions in 2012.

## Statistical analysis

### 2.1 Resource selection function

We used the amt package in R (Signer et al., 2019) to prepare the data and fit the resource selection function (RSF) and step selection functions (SSFs). We used the random_points() function to generate random locations (i.e., availability sample) within the minimum convex polygon (MCP, clipped to exclude land, note a buffer around the MCP can also be added), which by default generated 10 random locations per observed location. We used the extract_covariates() function to extract the corresponding prey diversity estimates for each observed and available location. We fit the RSF using the fit_rsf() function including the covariate for prey diversity (*prey*). fit_rsf()estimates the RSF coefficients by fitting a logistic regression to the use-available observations $\mathbf{y}=\{y_{1},\ldots, y_{i}, \ldots,y_{n}\}$, where $y_{i}∊\{0,1\}$. This is modeled using a Bernoulli distribution as:

Equation S 1

$y_{i}\sim$Bernoulli$(P(y_{i}=1|x_{prey,i}))$,

where we use a logit link function to keep the probability $P(y_{i}=1|x_{prey,i})$ between 0 and 1:

Equation S 2

$P(y_{i}=1|x_{prey,i})= \frac{\exp(\beta_{prey}x_{prey,i})}{1 + \exp(\beta_{prey}x_{prey,i})}$.

By default, R fits logistic models with an intercept, however, in the context of an RSF based on use-available data, this intercept is not interpretable. Therefore, the RSF derived from this model excludes the intercept:

Equation S 3

$w(x_{prey})=\beta_{prey}x_{prey}$ .

Since the prey diversity data was autocorrelated and independent data is required for RSFs, we also fit an RSF on a thinned dataset comprising of every 10^th^ location in the full dataset, using the same equations as the RSF on the full dataset (see tutorial).

We calculated the log of the relative selection strength (log-RSS) for prey diversity from our fitted models (Avgar et al., 2017; Fieberg et al., 2021), which is calculated as the relative frequency distribution based on the covariate value at location *a* relative to location *b*:

Equation S 4

$\frac{F^{U}(x_{prey,a})}{F^{U}(x_{prey,b})}=\frac{\exp(\beta_{prey}x_{prey,a}))}{\exp(\beta_{prey}x_{prey,b})}$ .

Further, we predicted the relative probability of use in space given our fitted models by applying equation S2 (without the intercept term, $\beta_{0}$) to each cell *j* in our study area, and normalizing the prediction following:

Equation S 5

$\frac{w(x_{prey,j})-min(w(\mathbf{x}_{prey}))}{max(w(\mathbf{x}_{prey}))-min(w(\mathbf{x}_{prey}))}$ ,

where $\mathbf{x}_{prey}$ is a vector that contains the prey diversity value for all cells in our study areas.

### 2.2 Step selection function

We used the steps() function to turn the dataset into “steps” (i.e., with a start and end location) rather than just observations. Next, we used the random_steps() function to generate 10 random steps per each observed step. The random_steps() function also generated a “step ID” column which represents each observed step and their control (random) steps (T = 140 observed steps). Control steps were created by sampling step lengths from a gamma distribution and turning angles from a von Mises distribution fit to the observed steps$.$ Similar to the RSF, we used the extract_covariates()function to extract the prey diversity estimate at the end of each observed and available step. Note that for simplicity in our tutorial, we just included the prey diversity covariate at the end of the step, but it could also be included at the start of the movement step using extract_covariates(fish_raster, where = “both”). We fit the SSF using the fit_clogit() function, including the covariate for prey diversity, the log of the step length, the cosine of the turning angle, as well as a strata() for step ID.

Using fit_clogit() to estimate SSF parameters requires that distributions of the movement kernel (i.e., step length and turn angle distributions) are of the exponential family, such that the product of movement kernel and habitat selection kernel can be defined in linear form. We used the gamma distribution for step length and von Mises distribution for turn angle, which defines the linear combination of SSF, $\eta_{t,i}$, predictors as follows (see Avgar et al., 2016 for full derivation):

Equation S 6

$\eta_{t,i}=w(x_{prey,i})\phi(l_{t,i},\theta_{t,i})$

$=\beta_{1}x_{prey, t,i}+\beta_{l}l_{t,i}+\beta_{ln}\ln(l_{t,i})+\beta_{\theta}cos\left( \theta_{t,i} \right)$.

As a computationally efficient method to approximate the integral in Equation 6 in the main text, we used a conditional logistic regression, where each observed location $s_{t,0}$ was coupled with m = 10 control points associated with control steps defined above, together, defining 11 potential locations $\mathbf{s}_{t,0:10}=\{s_{t,0}, ...,s_{t,10}\}$ at each time *t*. In the case of fitting the SSFs using amt, we consider each time *t* as a stratum (coded as strata(step_ID)) on the conditional probability of the observed step (Avgar et al., 2016; Michelot et al., 2023), which is defined as:

Equation S 7

$P\left( y_{t,i}=1 | {\mathbf{X}_{t},\mathbf{s}}_{t,0:10},\mathbf{s}_{1:t-1,0} \right)=\frac{\exp\left( \eta_{t,i} \right)}{\sum_{j=0}^{10} \exp\left( \eta_{t,j} \right)}$.

We also fit a SSF where we allowed prey diversity to affect the movement (specifically, the shape of the gamma distribution); thus, this model was fit similarly but included an interaction between prey diversity and the natural log of the step length:

Equation S 8

$\eta_{t,i}=w(x_{prey,i})\phi(l_{t,i},\theta_{t,i},x_{prey,i})$

$=\beta_{1}x_{prey, t,i}+\beta_{l}l_{t,i}+\beta_{ln}\ln\left( l_{t,i} \right)+\beta_{\theta}cos\left( \theta_{t,i} \right)+\beta_{2}x_{prey, t,i}\ln\left( l_{t,i} \right)$.

Note that the likelihoods between a conditional logistic regression and SSF are equivalent when the movement kernel and habitat selection function are modeled using a distribution from the exponential family. As such, when using a conditional logistic regression to fit a SSF, we are therefore limited to using distributions such as the exponential, gamma distributions, or half normal for step length, and von Mises distribution for turning angles (Avgar et al., 2016; Michelot et al., 2023). However, using a general model (not conditional logistic regression) to consider other distributions (e.g., Weibull for step length and/or wrapped Cauchy for turning angle) may improve model fit (Michelot et al., 2023).

Similar to the RSF, we calculated the log-RSS for prey diversity from our fitted models (Avgar et al., 2017; Fieberg et al., 2021). For spatial inference, we estimated the utilization distribution (UD) from each model. The UD is defined as the two-dimensional relative frequency distribution of space use of an animal (Van Winkle, 1975). We calculated the steady-state UD, which is defined as the long-term expectation of the space-use distribution across the landscape (Signer et al., 2017). To do this, we simulated an animal movement track with 10,000 locations that started at the first observed location (Fig. S3), and we removed the first 200 locations (2%) as a burn-in to reduce bias associated with setting the starting point. We obtained the steady-state UD using the hr_ud() function in amt which counts the number of times each cell was visited by the simulated animal, and normalized these frequencies to sum to 1 over the study area (Signer et al., 2017).

### 2.3 Hidden Markov model

We used the extract_covariates() function from the amt package to extract the prey diversity estimates for the observed data. We used the momentuHMM package in R (McClintock & Michelot, 2018) for the rest of the data preparation and to fit the hidden Markov model (HMM). We used the prepData() function to convert the data into “steps”, and to compute step lengths and turning angles for each step. We assumed the step lengths followed a gamma distribution and the turning angles followed a von Mises distribution. We initially fit a two-state HMM with prey diversity as a covariate in the transition probability (called hmm_trans_2 in the tutorial), using the fitHMM() function, and the getPar0() function to compute starting parameter values for each state, including the mean and standard deviation of step length distribution and the turning angle concentration. However, inspection of the histograms of step length and turning angle and pseudo-residual plots suggested an additional state was not being captured by the model (see Appendix 4). Therefore, we fit a three-state HMM, which improved the coverage in the histograms. We refit the three-state HMM using these new starting parameters (from getPar0()). The final model which was used in the main paper, a three-state HMM with prey diversity as a covariate on the transition probability (called hmm_trans_3 in the tutorial), was:

Equation S 9

$\gamma_{t,i,j}=\frac{\exp(\beta_{0,i,j}+\beta_{1,i,j}x_{prey,t})}{\sum_{l=1}^{N} \exp(\beta_{0,i,l}+\beta_{1,i,l}x_{prey,t})}$ ,

where $x_{prey,t}$is the prey diversity at time $t$, $N$ is the number of behavioural states (set to $N=3$ in the final model), and $\beta_{0,i,j}$ and $\beta_{1,i,j}$ are the intercept and coefficient that quantify the effect of prey diversity on transition probability from state $i$ to $j$, respectively. We fixed $\beta_{0,i,j} =\beta_{1,i,j}=0$ when $i=j$ (i.e., the diagonal of the transition probability matrix $\boldsymbol{\Gamma}$).

We also show in the tutorial how covariates can alternatively be included to modify the observation probabilities, that is, a three-state model including the covariate effects on the step length mean and turning angle concentration (Table S3 and S4, called hmm_obs_3 in the tutorial). This is typically used for factors that are unlikely to affect the probability of being in a behaviour (e.g., abiotic factors that would affect movement capacity), however, to demonstrate how this is done in our tutorial, we allowed prey diversity to affect the mean step length $\mu^{(l)}$ and turning angle concentration $\kappa^{(\theta)}$ for each state *i*:

Equation S 10

$ln(\mu_{t,i}^{(l)})=\beta_{i,0}^{(\mu^{(l)})}+\beta_{i,prey}^{(\mu^{(l)})}x_{prey,t}$ ,

Equation S 11

$ln(\kappa_{t,i}^{(\theta)})=\beta_{i,0}^{(\kappa^{(\theta)})}+\beta_{i,prey}^{(\kappa^{(\theta)})}x_{prey,t}$ .

Similarly, step length standard deviation could be modelled as a function of environmental covariates for each state *i*:

Equation S 12

$ln(\sigma_{t,i}^{(l)})=\beta_{i,0}^{(\sigma^{(l)})}+\beta_{i,prey}^{(\sigma^{(l)})}x_{prey,t}$ .

All behaviours were assumed to be correlated random walks with mean turning angle $\mu_{i}^{(\theta)}$fixed to 0. Finally, we show how they can also be included in both transition and observation probabilities simultaneously (called hmm_obstrans_2 and hmm_obstrans_3 in the tutorial), although this model is complex and perhaps not appropriate in cases where the state-dependent distributions have a lot of overlap.

We extracted the stationary state probabilities using the plotStationary() function, and we used the viterbi() function to decode the hidden states.

## References

Avgar, T., Lele, S. R., Keim, J. L., & Boyce, M. S. (2017). Relative selection strength: Quantifying effect size in habitat‐and step‐selection inference. *Ecology and Evolution*, *7*(14), 5322–5330.

Avgar, T., Potts, J. R., Lewis, M. A., & Boyce, M. S. (2016). Integrated step selection analysis: bridging the gap between resource selection and animal movement. *Methods in Ecology and Evolution*, *7*(5), 619–630.

Costa, D. P., Robinson, P. W., Arnould, J. P. Y., Harrison, A., Samantha, E., Hassrick, J. L., Hoskins, A. J., Kirkman, S. P., Oosthuizen, H., & Crocker, D. E. (2010). Accuracy of ARGOS locations of pinnipeds at-sea estimated using Fastloc GPS. *PLoS ONE*, *5*(1). https://doi.org/10.1371/journal.pone.0008677

Fieberg, J., Signer, J., Smith, B., & Avgar, T. (2021). A ‘How to’ guide for interpreting parameters in habitat‐selection analyses. *Journal of Animal Ecology*, *90*(5), 1027–1043.

Florko, K. R. N., Shuert, C. R., Cheung, W. W. L., Ferguson, S. H., Jonsen, I. D., Rosen, D. A. S., Sumaila, U. R., Tai, T. C., Yurkowski, D. J., & Auger-Méthé, M. (2023). Linking movement and dive data to prey distribution models: new insights in foraging behaviour and potential pitfalls of movement analyses. *Movement Ecology*, *11*(1), 17.

Florko, K. R. N., Tai, T. C., Cheung, W. W. L., Ferguson, S. H., Sumaila, U. R., Yurkowski, D. J., & Auger-Méthé, M. (2021a). Predicting how climate change threatens the prey base of Arctic marine predators. *Ecology Letters*, *24*, 2563–2575. https://doi.org/10.1111/ele.13866

Florko, K. R. N., Tai, T. C., Cheung, W. W. L., Sumaila, U. R., Ferguson, S. H., Yurkowski, D. J., & Auger-Méthé, M. (2021b). Predicting how climate change threatens the prey base of Arctic marine predators. *Dryad*, *dataset*. https://doi.org/https://doi.org/10.5061/dryad.x69p8czjs

Jonsen, I. D., Grecian, W. J., Phillips, L., Carroll, G., McMahon, C., Harcourt, R. G., Hindell, M. A., & Patterson, T. A. (2023). aniMotum, an R package for animal movement data: Rapid quality control, behavioural estimation and simulation. *Methods in Ecology and Evolution*, *14*(3), 806–816.

McClintock, B. T., & Michelot, T. (2018). momentuHMM: R package for generalized hidden Markov models of animal movement. *Methods in Ecology and Evolution*, *9*(6), 1518–1530. https://doi.org/10.1111/2041-210X.12995

Michelot, T., Klappstein, N. J., Potts, J. R., & Fieberg, J. (2023). Understanding step selection analysis through numerical integration. *ArXiv Preprint ArXiv:2308.15678*.

Signer, J., Fieberg, J., & Avgar, T. (2017). Estimating utilization distributions from fitted step‐selection functions. *Ecosphere*, *8*(4), e01771.

Signer, J., Fieberg, J., & Avgar, T. (2019). Animal movement tools (amt): R package for managing tracking data and conducting habitat selection analyses. *Ecology and Evolution*, *9*(2), 880–890.

Van Winkle, W. (1975). Comparison of several probabilistic home-range models. *The Journal of Wildlife Management*, 118–123.

# Appendix 3: Data for case study on ringed seals in Hudson Bay

[[github link](https://github.com/kflorko/movementstats_review/tree/main/data)]

# Appendix 4: Code tutorial for case study on ringed seals in Hudson Bay

[[github link](https://github.com/kflorko/movementstats_review/blob/main/script/Appendix3.Rmd)]
